# Icings and groundwater conditions in permafrost catchments of northwestern Canada

**DOI:** 10.1038/s41598-020-60322-w

**Published:** 2020-02-24

**Authors:** Hugo Crites, Steve V. Kokelj, Denis Lacelle

**Affiliations:** 1 Department of Geography, Environment and Geomatics, University of Ottawa, Ottawa, ON Canada; 2 Northwest Territories Geological Survey, Government of Northwest Territories, Yellowknife, NWT Canada

**Keywords:** Hydrology, Cryospheric science

## Abstract

Icings are sheet-like masses of ice that form on the ground surface or in fluvial channels from groundwater seepage. Although the presence of icings in the landscape is known, few studies investigated their regional distribution and explored relations with terrain factors including permafrost and winter baseflow conditions. Here, we mapped the distribution of icings in a 618,430 km^2^ area of northwestern Canada from a stack of 573 Landsat imageries (1985–2017) and determined using hydrometric data the winter baseflow contribution to the total annual discharge of 17 rivers in the study area. The 1402 mapped icings occur preferentially at the foothills of heavily faulted karstic mountainous regions in the continuous permafrost. Winter baseflow and its contribution to annual discharge was lower in continuous permafrost catchments than in discontinuous permafrost but showed a general increase over the 1970–2016 period. As such, the distribution of icings appears to be sensitive to winter air temperatures and winter baseflow conditions and icings located at the southern boundary of continuous permafrost would be more sensitive to degrading permafrost and the predicted increase in winter baseflow.

## Introduction

Permafrost and hydrology are coupled components in many Arctic systems^1–4^. In northwestern Canada and Alaska, warming climate has caused an increase in active layer thicknesses and permafrost temperatures^5–7^, which have affected hydrological processes. For example, the recent reduction in the area of ponds and lakes in discontinuous permafrost has been associated with an increase in sub-subsurface drainage and connectivity^8,9^. Additionally, despite little change in total annual discharge^10,11^, there is evidence from gauging stations of an increase in winter baseflow for many rivers^12–14^. These changing permafrost and groundwater conditions may affect seasonal ice formation from the freezing of groundwater seeping to the surface such as icings, also known as naled or aufeis. The sustained groundwater flow that form these features provide critical habitats for overwintering of certain fish species, including the Dolly Varden char^15–17^ and are a source of perennial drinking water for some northern communities^18^. Icings can also store large amounts of ice that progressively melts during the summer and recharge local streams and rivers long after the melt of late-lying snowbanks.

Icings are horizontal to sub-horizontal sheet-like ice mass that develop during winter on the surface by the freezing of groundwater that repeatedly or continuously seep from the supra-permafrost (ground icings), from a spring (spring icings), or that emerges from below the river ice (river icings)^19–22^. Icings have been reported in all Arctic regions^23–28^ and studies have focused on their morphological characteristics^22^, their development and energy balance^22,29,30^, their seasonal and perennial contributions to fluvial discharge^31^ and groundwater recharge conditions^18,32^. Spring icings commonly form in places where the perennial groundwater flow through a talik is forced to the surface by a reduction in aquifer permeability due to permafrost that impedes sub-surface flow^19,22,33^. In similar fashion, river icings form in places along fluvial channels where baseflow is sufficiently restricted by the freezing along a cross-section of the channel caused by either a change in channel gradient or in thickness of alluvial sediments^19,22,33^. Spring and river icings tend to occur at the same location year after year, generally with the same shape, and to be larger than ground icings which have a more random spatial and temporal recurrence in the landscape^19,22,33,34^. Given the strong linkage between icings and groundwater flow, insights on the response of icings under degrading permafrost and changing groundwater conditions may be gained by investigating their distribution in the landscape and relations with terrain and winter discharge conditions.

The objectives of this study are to: (1) map using a semi-automated approach the distribution of spring and river icings in northwestern Canada from a dense stack of Landsat imageries (1985–2017); (2) determine using the Environment Canada historical hydrometric data the winter baseflow contribution to the total annual discharge of rivers in the study area and their temporal trends (1970–2016). Based on the results, relations between the distribution of icings, terrain and winter baseflow conditions were explored which can inform about the response of icings to changing permafrost and groundwater conditions.

## Study area

**Figure 1:**
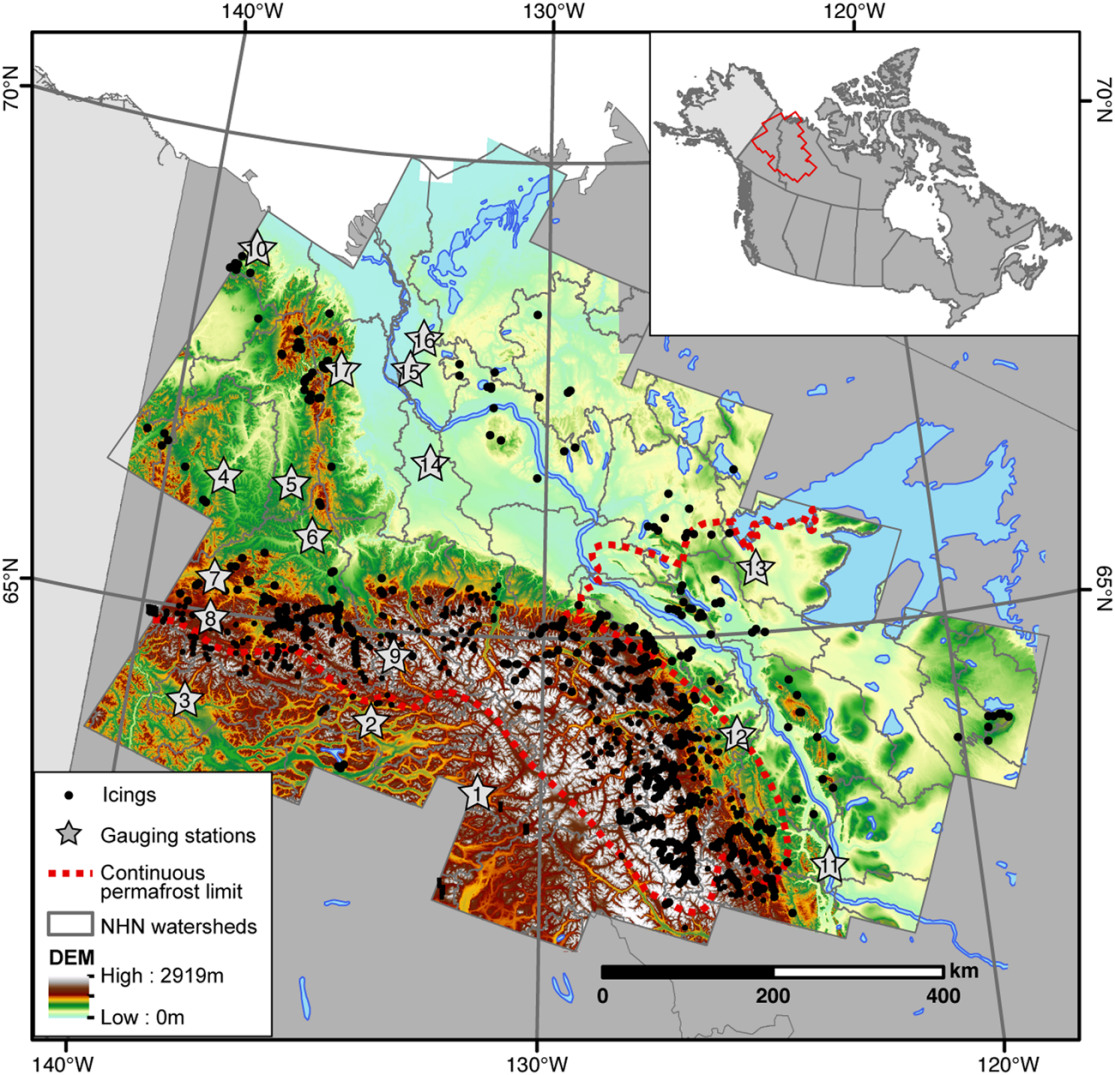
Icing occurrence in northwestern Canada. The icings were identified using a semi-automated approach and a dense stack of Landsat imageries. Active gauging stations and level-3 watersheds are also showed (see Table 1 for gauging station names). Digital elevation model (DEM) background derived from 30 m Canadian Digital Elevation Data (www.geogratis.ca). Map generated using ArcGISv10.

The study region in northwestern Canada ranges from 62 to 69°N and from 118 to 140°W; covering an area of about 618,430 km^2^ (Fig. 1). Elevations range from sea level along the Beaufort Sea coastline to 2920 m a.s.l. in the Mackenzie Mountains, with nearly 50% of the study area located below 400 m a.s.l. A total of 28 ecoregions are found within the study area; the northern sector includes low-lying alluvial and glaciated terrain (Mackenzie Delta and various plains), fluvially-incised moraines (Peel Plateau) and the Richardson and Brooks mountain ranges. The southern sector is more mountainous and includes the Mackenzie, Selwynn and Ogilvie mountains. The geology of the study area is predominantly composed of lower Cretaceous sandstone-conglomerate-shale and siliclastic rocks, Jurassic shale-siltstone, Carboniferous limestone-dolomite, Permian clastic and carbonate rocks and Cambrian to Silurian limestone^35^. The surficial geology is quite varied with mainly fine-grained glacial deposits in glaciated regions and colluvial deposits at the bottom of hillslopes; colluvium and bedrock are commonly found at higher elevation^36,37^. Vegetation range from tundra in the northern plains and mountainous regions to boreal forest in the southern region. Wetlands and peatlands are also common features on poorly drained lacustrine sediments and become more common southward in the Mackenzie Valley^38^.

The climate in the study area is characterized by a subarctic climate regime with cold winters and cool summers. The nine climate stations in the study area report mean annual air temperature (MAAT) for the period 1980–2010 ranging from near −0.7 °C at Fort Simpson in the southern region to near −10.5 °C at Tuktoyaktuk in the north. The temperature conditions throughout the mountainous region are not well documented; however temperature inversions leading to colder winter temperatures in the valley bottoms probably occur^39^. Since 1980, the northern region has experienced a warming trend of 0.07 to 0.11 °C yr^−1^, whereas the more southern region experienced a warming of 0.01 to 0.07 °C yr^−1^; with four stations showing a significant trend at <0.1 level (Table S1). Total annual precipitation across the study area range from 240 to 440 mm with the southern region receiving more precipitation. Since 1980, no significant trends in precipitation at the <0.1 level was observed across the study region (Table S1).

Permafrost is continuous in the northern plains and higher elevations in mountainous regions (55% of study area)^40,41^. Extensive discontinuous permafrost occurs at lower latitudes and elevations (40% of the study area)^40,41^. Mean annual ground temperatures (2013–2015) in the discontinuous permafrost zone ranged between −2 and 0 °C with colder temperatures in the continuous permafrost zone (−6 to −4 °C). Permafrost temperatures between 2007 and 2015 along the Wrigley and Fort Good Hope corridor (63 to 66°N) showed an increase of 0.02–0.03 °C y^−1^ while further north in the Mackenzie valley changes in ground temperature have been closer to 0.1 °C per year^42,43^.

The study area includes 55 level-three watersheds and most of the rivers are characterized by sub-arctic to arctic nival flow regime. Gauging records of 23 rivers in NWT over the 1977–2007 period showed that winter baseflow contributes 1.2–87% to the total annual discharge, with the majority (20 out of the 23 stations) experiencing an increase in winter baseflow contribution (0.5–271% yr^−1^)^14^. Similar observations between 1949 and 2005 were made for 21 gauging stations in the Yukon River basin where groundwater contributed 4.7–47.4% of the total annual discharge and with an increase of 0.4–2.6% yr^−1^^12^. It was suggested that the increase in groundwater contribution was attributed to permafrost degradation in response to warming air temperature^12^.

The presence of permafrost largely restricts groundwater recharge to places where fractured bedrock, sinkholes or dissolution channels are exposed at the surface^44^. In the central Mackenzie Valley (Smith Creek, White Sand Creek and Gayna rivers) and northern Yukon (Fishing Branch and Firth rivers), groundwater is recharged mostly from Fall precipitation through saturated organic soils with no advection of heat to the subsurface^32,45^. A similar recharge system has also been described for the North Klondike watershed in YT^46^. Based on noble gas and isotope geochemistry, groundwater circulation times in the study area were found to be in the order of two to three decades^45^. The groundwater mostly discharge through overburden but direct discharge via fissures in exposed bedrock and talus has been observed where bedrock intersects the surface due to folding or if the unit is intersected by a fault. Although folding is the predominant structural style in the Mackenzie Mountains, thrust faults remain important for groundwater flow^47^.

## Results

### Distribution of icings in northwestern Canada

**Figure 2:**
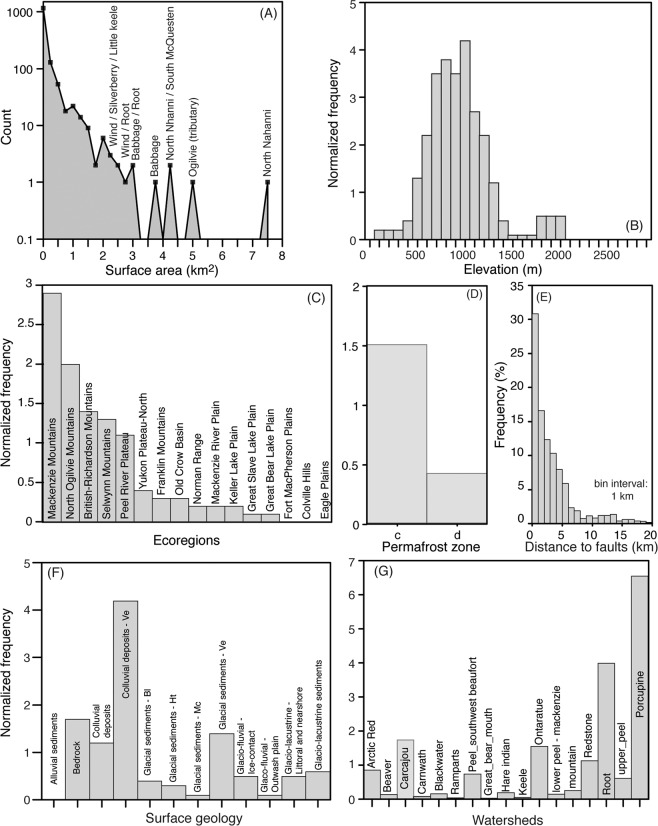
Frequency distribution of mapped icings in northwestern Canada**. (A**) Surface area of icings. (**B**) Histogram showing the normalized frequency distribution of icings with respect to elevation in study area in 100 m elevation bins. (**C**) Histogram showing the normalized frequency distribution of icings with respect to ecoregions. (**D**) Histogram showing the normalized frequency distribution of icings with respect to permafrost zones (c = continuous; d = discontinuous). (**E**) Histogram showing the distance between icings and the closest geological fault in 1 km bin interval. (**F**) Histogram showing the normalized frequency distribution of icings with respect to surficial geology. (**G**) Histogram showing the normalized frequency distribution of icings with respect to watersheds.

In the study area, a total of 1402 icings with >30% annual occurrence between 1985–2017 were identified (although most occurred annually) (Fig. 1). Known icing occurrences in Tombstone Territorial Park and in northern YT and adjacent NWT were identified in the mapping (i.e., ref. ^24^). Additionally, a comparison with late-winter high-resolution imagery confirmed the presence of icings in the Sahtu highlands and on the Hare Indian river (Fig. S1). The average surface area of the icings was 0.2 km^2^ and a total of 19 icings had surface areas greater than 2 km^2^ (Fig. 2a). These mega icings account for 21% of the total icing area in the mapped region. The cumulative surface area of icings is 277 km^2^ and accounts for 0.04% of the study area. Due to the pixel resolution of the Landsat images (30 m), features <0.0036 km^2^ were likely not mapped.

Icings were found at elevations ranging between 15 and 2022 m a.s.l. (Fig. 2b). Normalizing the frequency count of icings by the frequency distribution of elevation in 100 m bins reveals that icings are more likely to occur at elevations of 500−1300 m by factors of 1.2 to 4.2. The highest icing count was found in the Mackenzie Mountains, which contained more than half of the total icings identified (n = 718; Fig. S2). When the frequency of icing count is normalized by the frequency distribution of area covered by the ecoregions, the icings are more likely to occur in mountainous regions by factors of 2.9 to 1.3: the Mackenzie (factor of 2.9), followed by the North Ogilvie (2.0×), British-Richardson (1.4×) and Selwynn mountains (1.3×); the plains contained the lowest likelihood of icing occurrence (<0.3×). The majority of icings occur in association with areas of carbonate, sandstone and evaporite bedrock; few are found in volcanic rocks. The majority of icings (n = 1179) were within 6 km of the nearest major fault system: average distance being 5 km and only 25 icings were found at distances of 20–207 km, the farthest distance between icing and fault (Fig. 2e). Most of the icings (n = 1174) were found in the continuous permafrost zone (Fig. 2d). Normalized to the frequency distribution of permafrost zones, icings are more likely to occur in continuous permafrost by a factor of 1.5. In terms of type of surficial geology, icings are more likely to occur by factors of 1.2 to 4.2 on undifferentiated bedrock, colluvial mass-wasting deposits (both veneer and undifferentiated) and veneers of glacial sediments (Fig. 2f).

**Figure 3:**
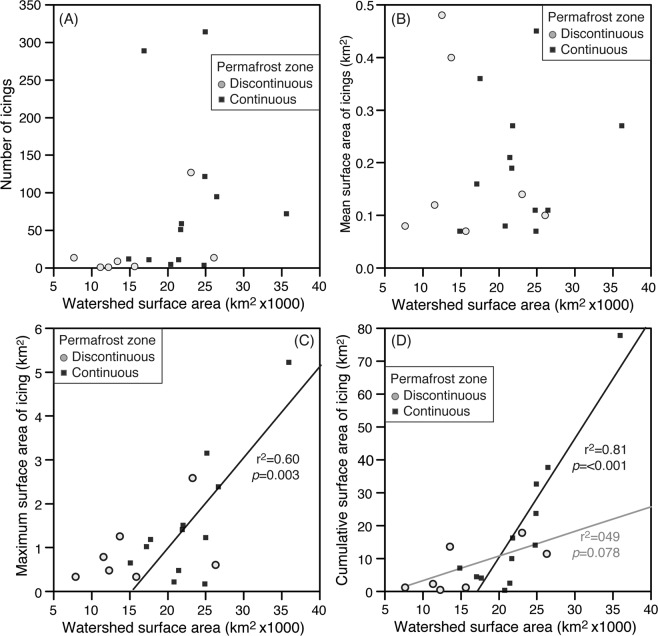
Number and extent of icings within their watersheds. Scatter plots of watershed surface area relative to (**A**) number of icings in the watershed; (**B**) mean surface area of icings in the watershed; (**C**) maximum surface area of icing in the watershed; (**D**) cumulative surface area of icings in the watershed. Linear trend lines are shown for statistically significant relations (*P* < 0.1).

Icings occurred in 17 of the 55 watersheds in the study area (Fig. 2g). Icings were all found in watersheds with extents <26,200 km^2^ in the discontinuous permafrost and <35,800 km^2^ in continuous permafrost. When normalized by the frequency distribution of area covered by the watersheds, icings are more likely to occur in the upper Porcupine, Root, Carcajou, Ontaratue and Redstone watersheds by factors of 6.5 to 1.1. These five watersheds represent 29% of the study area and contain 78% of the icings. The larger icings in the study area were observed on the North Nahanni river (7.7 km^2^), on a tributary of the Ogilvie river (5.2 km^2^) and on the Babbage river (3.8 km^2^); however, the largest icing in northwestern Canada is the upper Firth river icing (~31 km^2^)^31^. The number of icings and their mean surface area showed no relation with their watershed extent (Fig. 3). However, the maximum and cumulative surface area of icings were positively correlated with their watershed extent, with relations being significant for the continuous permafrost zone (Fig. 3). Since the groundwater feeding the icings is recharged from precipitation received within the watershed, the relation with total volume of precipitation in the watershed and icings was explored. With total precipitation, the significance of the positive relation improved between the maximum and cumulative surface area of icings in continuous permafrost catchments (Fig. S3). Although the thickness of the icings could not be determined, it generally varies between 2–3 m^24,31^. Using an ice thickness of 2 m, an average of 0.33 ± 0.28% of total annual precipitation within the watersheds is stored in icings, and it increases to 0.51 ± 0.44% if a thickness of 3 m is used.

### River discharge in northwestern Canada

**Figure 4:**
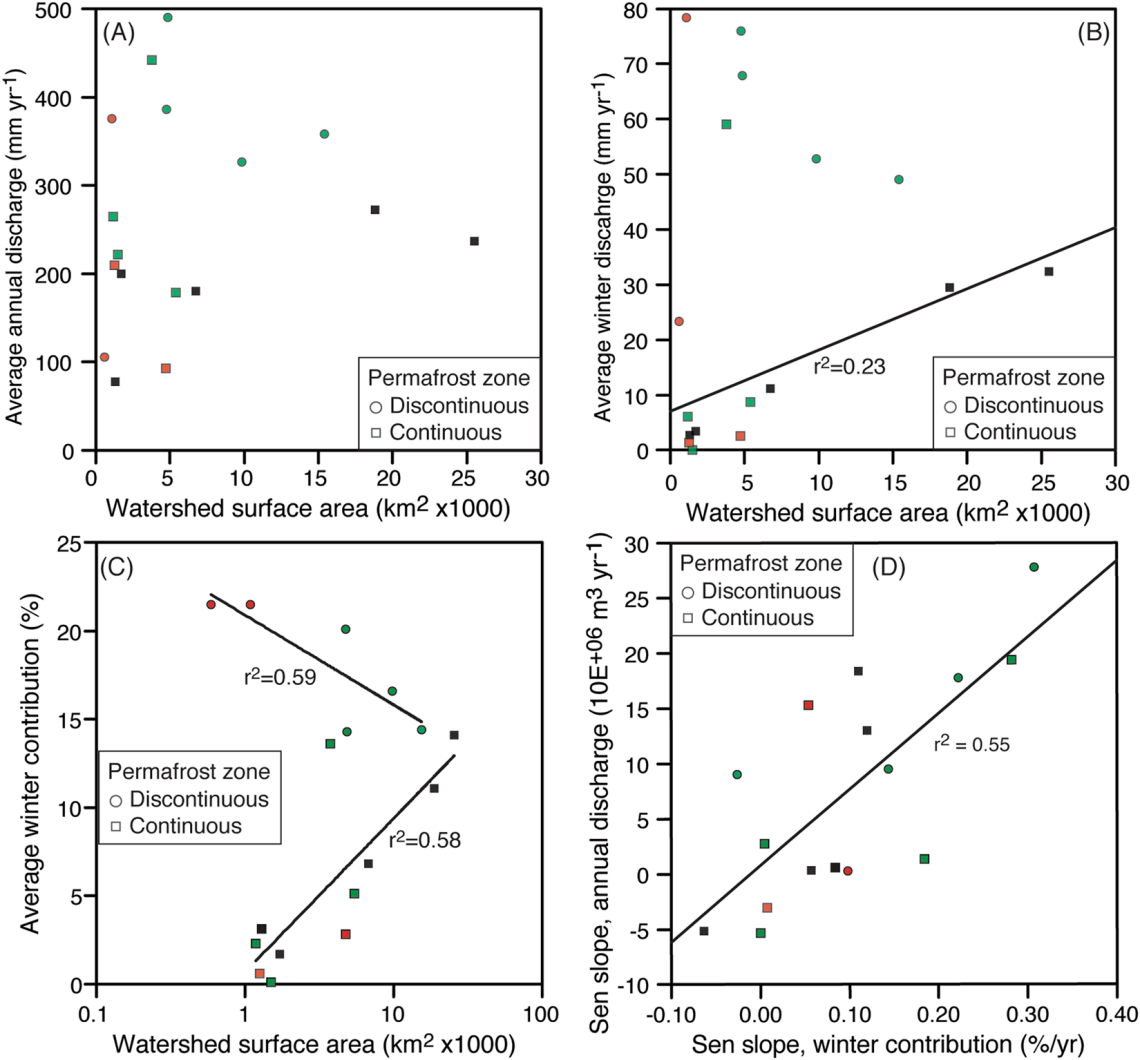
Hydrometric data of gauging stations in northwestern Canada separated by permafrost zone. (see Table 1 for list of stations). (**A**) Relation between annual discharge and watershed area in continuous and discontinuous permafrost. (**B**) Relation between winter discharge and drainage area in continuous and discontinuous permafrost. (**C**) Relation between winter discharge contribution to total annual discharge and drainage area in continuous and discontinuous permafrost. (**D**) Relation of Sen slope trend between total annual discharge and winter contribution. Note: Red symbol = no icings in the watershed; Green symbol = icings along the river with a gauging station; Black symbol = icings in the watershed but not along the river on which the gauging station is found. Linear trend lines are shown for statistically significant relations (*P* < 0.1).

Out of the 17 gauging stations in the study area, 13 are situated in watersheds that contain icings and 8 of them are situated along a river downstream of at least one mapped icing. The total annual discharge of the 17 rivers range from 77 to 490 mm yr^−1^ (or 6.2 ×10^^7^ to 6.1 ×10^^9^ m^3^ yr^−1^) (Fig. 4a). The winter baseflow range from 0.06 to 78.5 mm yr^−1^ (or 8.4 ×10^^4^ to 8.3 ×10^^8^ m^3^ yr^−1^) with gauging stations situated in the continuous permafrost generally have lower winter discharge (Fig. 4b). With the exception of one gauged river, the winter baseflow at stations in continuous permafrost was <30 mm yr^−1^, which is nearly half of the stations in discontinuous permafrost.

**Figure 5:**
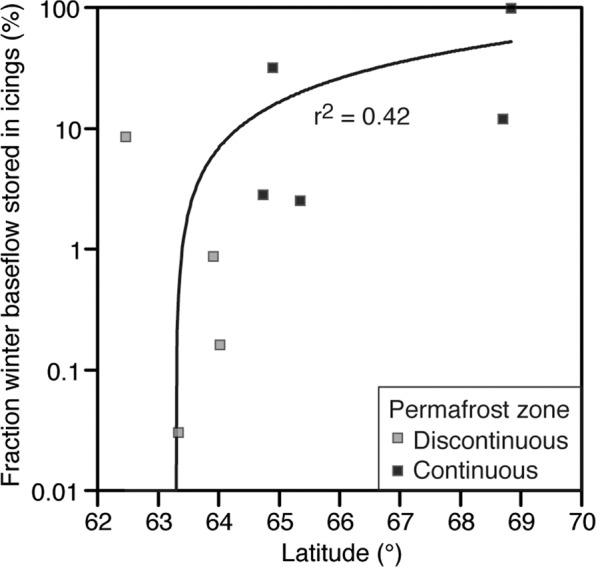
Fraction of winter baseflow stored in icings. The volume of water stored in the icings that is derived from winter baseflow contribution to total annual discharge was calculated for the eight icings (out of the 1402 ones) that have a gauging station along the river. The calculation is corrected for volume of water stored in the icings and assumes icing thickness of 2.5 m. The fraction of winter baseflow stored in icings (Iwb) was calculated from: ${I}_{wb}( \% )=({I}_{sa}\times {I}_{t})/(\frac{{D}_{w}}{{D}_{a}}+{I}_{sa}\times {I}_{t})\times 100$ where, Isa = surface area of icing (m^2^), It = thickness of icing (m), Dw = winter discharge (m^3^), Da = total annual discharge (m^3^).

The average winter baseflow contribution to total annual discharge at the 17 gauging stations over the individual periods of record ranged from 0.1 to 22% (Table 1). The Babbage, Rat and Eagle rivers had the lowest winter contribution (<2%), two of which are north of the Arctic circle, whereas two of the southerly rivers, the Beaver and North Klondike, had the highest (20–22%). The average winter baseflow contribution to total annual discharge showed distinct trends with respect to permafrost zone. Rivers located in the discontinuous permafrost zone had the highest winter contribution (14–22%) but showed a significant negative trend with watershed extent (Fig. 4c). By contrast, the average winter baseflow contribution to rivers located in the continuous permafrost zone ranged from 0.1 to 14.1% and showed a significant positive trend with watershed extent (Fig. 4c). The volume of water stored in the icings that is derived from winter baseflow can be evaluated for the eight icings (out of the 1412 ones) that have a gauging station along the river. Assuming icings thickness is 2.5 m, the fraction of winter baseflow stored in the icings range from 0.03 to 98% (average = 18.2%; median = 2.5%); the Babbage River watershed has the highest fraction due to the low winter contribution to total annual discharge and numerous icings (Fig. 5).

**Table 1:** Gauging stations in study area, winter contribution to total annual discharge, and trend analysis results. Map ID corresponds to gauging stations on Fig. 1.

Map ID	Station ID	Station name	Period of record	Drainage area (km^2^)	Annual discharge (mm)	Winter contribution (%)	Sen slope, winter contribution (% yr^−1^)	Sen slope, annual discharge (m^3^ yr^−1^)
1	09DA001	Hess River above Emerald Creek	*1977*–*2016*	4840	491	14.3	0.144+	9.5E + 06
2	09DB001	Beaver River below Matson Creek	*1996*–*2016*	4770	387	20.1	0.222	1.8E + 07
3	09EA004	North Klondike River near the mouth	*1975*–*2016*	1090	377	21.5	0.098	3.1E + 05
4	09FA001	Whitestone River near the mouth	*1979*–*2016*	6730	180	6.8	-0.063	−5.2E + 06
5	09FB002	Eagle River at Dempster Highway bridge	*1945*–*2016*	1720	200	1.7	0.057+	3.5E + 05
6	10MA001	Peel River above Canyon Creek	*1970*–*2016*	25500	237	14.1	0.110*	1.8E + 07+
7	10MA002	Ogilvie River at km197.9 Dempster Highway	*1975*–*1996*	5410	179	5.1	0.005	2.7E + 06
8	10MA003	Blackstone River near Chapman Lake airstrip	*1984*–*1995*	1180	265	2.3	0.046	1.9E + 06
9	10MB004	Bonnet Plume River above Gillespie Creek	*1981*–*1994*	3760	442	13.6	0.282	1.9E + 07
10	10MD002	Babbage River below Caribou Creek	*1978*–*1994*	1500	221	0.1	0	−5.3E + 06
11	10GA001	Root River near the mouth	*1975*–*2016*	9820	327	16.6	0.307***	2.8E + 07*
12	10HB005	Redstone River 63 km above the mouth	*1980*–*1995*	15400	359	14.4	−0.026	9.0E + 06
13	10JD002	Whitefish River near the mouth	*1978*–*1991*	4740	93	2.8	0.054	1.5E + 07
14	10LA002	Arctic Red River near the mouth	*1974*–*2016*	18800	272	11.1	0.120***	1.3E + 07
15	10LC003	Rengleng River below highway no. 8	*1976*–*2016*	1300	78	3.1	0.084**	6.1E + 05
16	10LC007	Caribou creek above highway no. 8	*1975*–*2016*	590	106	21.5	0.974***	7.2E + 05*
17	10MC007	Rat River near Fort McPherson	*1981*–*1990*	1260	210	0.6	0.008	−3.0E + 06

Level of significance of trend: + (0.1) *(0.05) **(0.01) ***(0.001).

Most discharge records showed an increase in winter baseflow contribution to the total annual discharge over the 1970–2016 period (Table 1). Out of the 17 rivers, 7 of them revealed a significant increase in winter contribution; only the Whitestone and Redstone rivers showed a non-significant decreasing trend (Table 1). In regards to total annual discharge, only the Root River and Caribou Creek showed a significant increase in total annual discharge; 12 rivers showed a non-significant increasing trend; the other three rivers showed a non-significant decreasing trend. In general, change in winter contributions were positively correlated with changes in total annual discharge; if one outlier is removed (Caribou Creek), the relation is significant at the 0.01 level (Fig. 4d).

## Discussion

Mapped spring and river icings in the study area occur preferentially at the foothills of heavily faulted karstic mountainous regions in the continuous permafrost zone (Figs. 1–2). Karstic terrain favours the recharge of groundwater system through sinkholes and dissolution channels^44^, and the proximity to fault zones provides flow paths and seepage points facilitating surface discharge through during the winter season^47^. Although the absence of detailed investigations precludes confirmation that each of the icing is associated with a nearby groundwater spring, the late winter high-resolution satellite imageries show that the Babbage, north Klondike, Blackstone, Hare Indian rivers have a perennial springs that starts upstream of the icings (Fig. S1). A similar relation between major fractures systems and distribution of icings was found in stream bottoms of the Shawnee Peak massif along the Alaska-Yukon border^48^. In this region, the Occidental Creek valley had one the most extensive laterally fractured zones and contained the thickest and most extensive icing formation.

Icings occur preferentially in continuous permafrost catchments and their maximum and cumulative surface area showed significant positive relations with watershed extents (Figs. 2–3); a similar relation was observed in northern Yukon and northeast Alaska^22,31^. Icings tended to be found in continuous permafrost catchments because the low winter air temperatures, presence of frozen ground and winter baseflow conditions favours their development. In the study area, the freezing-degree days ranges from about 3700 in the southern region to about 4500 in the northern region (ref. ^49^ https://climateatlas.ca/). As such the winter air temperature in the continuous permafrost zone increases length and severity of the freezing season and the likelihood that baseflow will be impeded in braided fluvial channels allowing for the development of icings^24,47^. It has also been suggested that the persistence of icings during the summer can cause lower ground temperatures in their vicinity than the surrounding landscape, which may create a positive feedback, promoting the development of icings during the following winter^22^. The lower abundance of icings in discontinuous permafrost catchments cannot be explained by the absence of perennial springs since many springs have been identified in the discontinuous permafrost zone^50^. However, the rivers in the discontinuous permafrost zone typically have higher winter baseflow and winter contribution to total annual discharge relative to those in continuous permafrost (Fig. 4). It is thus plausible that higher winter baseflow combined with convective heat exchange of the flowing water and a lower freezing-degree days prevents freezing of the river to its bed to impede baseflow. The absence of icings during years with higher winter baseflow was observed along the Fishing Branch by the inhabitants of Old Crow (i.e., ref. ^24^). A first order assessment of the winter baseflow conditions that support the development of spring and river icings can be made from the winter discharge in their respective watersheds. Our results suggest that icings are generally found in continuous permafrost watersheds with: (1) winter flow <60 mm yr^−1^; and (2) winter contribution to total annual discharge <20% (the two gauging stations with winter contribution >20% do not have icings). The Firth River icing had an calculated winter contribution of 20% to total annual discharge^31^, similar to our regional assessment. Although these differences are based on the available gauging stations, it does support the observation that the growth of icings require winter flow to be sufficient to prevent complete freezing along the fluvial channel but not high enough to maintain an open channel and prevent the total freezing of a cross-section along the channel. Under these circumstances, icing development within specific channel and streamflow contexts would be sensitive to increasing winter air temperatures and baseflow conditions.

In northwestern Canada, winter baseflow has increased for most rivers since 1970s (Table 1). Different inter-related factors may contribute to this trend. Spectral analysis of detrended data revealed that winter discharge of rivers respond to the solar (11-yr) and PDO-AO cycles (3–5-yrs) as it relates to their connection with precipitation^51–53^ (Fig. S4); a trend also reported by refs. ^46,54^ for rivers in the Ogilvie Mountains. The potential influence of solar and PDO-AO cycles on winter baseflow conditions relates to increasing groundwater recharge following multi-year increases in precipitation (e.g., refs. ^51,55^). However, cross-correlation of winter discharge with sun spot number, PDO and AO revealed that only a few stations had significant relations at the <0.1 level, which may be attributed to a non-linear response of precipitation, groundwater recharge and winter discharge. Refs. ^12,14^ also suggested that permafrost degradation in the study area is leading to increased groundwater contribution to total annual discharge in river systems. Based on projected warming scenarios, simulations revealed that winter contribution and flow would increase substantially with decreasing permafrost extent^13^; a change caused by deeper flow paths of supra-permafrost water, expansion of taliks and increasing hydraulic conductivity as permafrost soils approach 0 °C (e.g., refs. ^56,57^). However, changing groundwater flow was non-linear and most increase is predicted as permafrost extent decrease from 100 to ~70%^13^. Therefore, icing conditions along the boundary of continuous and discontinuous permafrost would be the most affected by degrading permafrost and the potential increase in winter baseflow discharge. In the mapped area in northwestern Canada, 84 icings (6%) in the continuous permafrost zone are found with 10 km of that boundary and 564 icings (40%) are within 50 km.

The findings of this study suggest that the distribution and growth of icings are sensitive to permafrost type, winter air temperatures and winter baseflow conditions. Icing conditions at the southern boundary of continuous permafrost should be most sensitive to degrading permafrost and the predicted increase in groundwater discharge. A northward shift in the continuous permafrost boundary would likely lead to a reduction in icing occurrences or perhaps a shift in their distribution if conditions in more northerly regions become more conducive to icing development. Considering that icings store on average 18% of winter baseflow contribution to total annual discharge of rivers, with an increasing storage trend along a latitudinal gradient (Fig. 5), a change in icings conditions will also affect hydrological conditions of local streams and rivers along which they are situated.

## Methods

### Mapping of icings

To map the distribution of spring and river icings in northwestern Canada, 573 level-2 Landsat images acquired between 1985–2017 and corrected for possible anisotropy in reflectance distribution created by large and mountainous areas were obtained from ESPA level-2 (https://espa.cr.usgs.gov). All images selected for analysis had a low cloud coverage (≤ 20%) and acquisition dates between May 1^st^ and June 30^th^. The late spring-early summer corresponds to a period when most of the snow has melted from the landscape and icings are more easily identifiable on the imagery. The list of path/row Landsat scenes is provided in Table S2.

The methodology uses a dense stack of Landsat 5 TM Landsat 7 ETM + imageries and follows a semi-automated approach established around Yellowknife (NWT)^34^, but modified for the study area with varied physiographic characteristics (Fig. S5). The mapping was done in ArcGIS 10.5 using *Python* scripts and involved a series of steps using the Landsat LT2 images. The Normalized Difference Snow Index (NDSI) was first calculated to discriminate snow, ice and water from bare soils and clouds. The Maximum Difference Ice Index (MDII) was then calculated on the thresholded-NDSI rasters to differentiate ice, water, snow and wet marl. Following these two steps, late-lying snowbanks at higher elevations in the Mackenzie Mountains, the Ogilvie Mountains, British-Richardson Mountains and the Selwynn Mountains were still being classified as ice. The snowbanks at high elevation were thus removed from the classification by creating a Topographic Position Index (TPI), a measure of terrain ruggedness and local elevation index, that defines hilltops, slopes and valley bottoms. The TPI values between < −5 and > +5 were used to mask late-lying snow on hilltops and slopes (mainly north-facing slopes). The approach was successful in removing most of the snow; however, snow still remained along some slopes and elevated plateaus. As icings tend to develop in valley bottoms along stream/river channels^19^, we thus created a slope mask (slopes>30°) to remove these snow patches. Following the application of the TPI and slope mask, mainly late-lying spring to early summer ice remained on the imageries; some noise was removed manually. Finally, the lake and river ice was removed using a Normalized Difference Water Index (NDWI) from mid-summer cloud-free scenes^58^. Pixels classified as water were digitized into polygons shapefiles using the *Raster to Polygon* tool. However, multiple braided rivers are present in the mountain ranges located west of the Mackenzie River and these can contain icings. Therefore, the NDWI was only applied to the flatter area east of the Mackenzie River. In addition, topographic shadows were being classified as water bodies by the water index. However, when exploring the pixel values of shadows and water bodies in the thermal band, values were much higher for water bodies than in the shaded areas. Hence, a thresholding value>105 in the thermal band was selected to remove shadows from the water mask. Finally, processes that are not icing-related are sometimes responsible for substantial snow and ice accumulation along the shorelines of rivers and lakes past the snowmelt period. This remnant ice and snow was eliminated from the classification by growing the water mask outwards by 1.5 times the pixel resolution^34^.

Following these steps, each scene resulted in a binary raster; where 0 indicated “no ice” and 1 indicated ice. To calculate icing recurrence, a raster containing the total image count for a given Landsat extent was created and used to normalize the dataset across the study area as the number of scenes varied between Landsat extents. To obtain percentage of recurrence, binary rasters from a stack were added together using the *Raster Calculator* and then divided by the amount of scenes in that stack. Using the *Raster to Polygon* tool, icing polygons were then created from the recurrence map; pixels with a value>30% were selected (note, the option *Simplify Polygons* was not selected in order to retain the pixel’s edge). Polygon attributes such as centroid, coordinates surface area were calculated with the *Calculate Geometry* function.

The identification of spring and river icings is limited by the pixel resolution of the Landsat images (30 m) and features <0.0036 km^2^ are likely not mapped. To validate the identification and extent of icings from the semi-automated approach, the results were compared to: (1) known location of icings in the study area (e.g. ref. ^24^); and (2) late-winter high-resolution imagery from WorldView-2 and GeoEye-1 (0.46 m and 0.41 m multispectral resolution, respectively). Although the presence of substantial snow cover throughout the river valley can conceal river ice, icings can be easily distinguished by its blueish color^59^.

### Spatial control on icing distribution

Relations between the spatial distribution of icings, elevation, ecoregions, permafrost zone, surface geology, bedrock geology, proximity to faults, and watershed area and precipitation were investigated in ArcGIS using the centroid of each icing and the *Extract Multi Values to Points* tool. Elevations were derived using ArcGIS Spatial Analyst using a mosaicked 30 m digital elevation model (CDED). The level-3 watersheds were derived from the the National Hydro Network *GeoBase Series* 1:50,000 scale watershed data. The CanBPv0 blended precipitation data from meteorological station data and satellite estimates was used to calculate total amount of precipitation received in each watershed^60^. The distribution of permafrost in the study area was derived from the permafrost and ground ice map of Canada (Geological Survey of Canada Bulletin 548; 10.4095/212210). Digital data of surficial geology from the Canadian Geoscience Map 195 (10.4095/295462). Geological faults and parent bedrock were derived from the Geological Survey of Yukon (http://www.geology.gov.yk.ca/) and Northwest Territories Geological Survey (https://datahub-ntgs.opendata.arcgis.com/) and merged to cover to the study area. The proximity of icings to faults was assessed using the *Near-dist* tool that calculates shortest distance between each single icing point and the nearest fault. To determine whether the frequency distribution of icings reflects the frequency distribution of terrain factors in the landscape, or if some components are associated with an enhanced probability of icing occurrence, the icing frequency was normalized by the frequency distribution of terrain factors.

### River discharge records

River and spring icings form during winter along sections of fluvial channels that typically freeze to the bed, which confines baseflow and generates hydrostatic conditions for groundwater to seeps to the surface^19–21^. As such, the discharge records from 17 rivers with watershed extent <25,000 km^2^ in the study area were analyzed for trends in total annual and winter discharge (Water Survey of Canada’s Hydrometric Database; http://www.wsc.ec.gc.ca/). Excluding the gauging stations along major rivers in the study area (i.e., Mackenzie, Yukon, Peel rivers) because icings do not develop along them, the selected stations represent ~70% of the year-round active stations; the others had discontinuous seasonal records only. Mean monthly discharge data was available for most years between 1970 and 2016 (Table 1). All 17 rivers develop an ice cover during the winter with baseflow sustained by groundwater. Total annual discharge and groundwater contribution during the winter months (December 1 to March 31) were converted to m^3^ yr^−1^ and mm yr^−1^ Analyses of long-term trends in total annual discharge and winter discharge contribution were performed using the non-parametric Sen slope with the significance of the trend tested with the Mann-Kendall test (i.e., refs. ^10,12^). This approach allows for missing data and the data does not need to conform to any distribution. Spectral analysis was performed on detrended annual and winter discharge to identify significant cycles related to solar activity and pacific decadal oscillation.

## Supplementary information

Supplementary Data.

Supplementary Data1.
