# Supplementary material for: Icings and groundwater conditions in permafrost catchments of northwestern Canada
Source: Sci Rep. 2020 Feb 24;10:3283. doi: 10.1038/s41598-020-60322-w (PMC7039954; doi:10.1038/s41598-020-60322-w)
Supplement: Supplementary file 1 — Supplementary Data. [file 41598_2020_60322_MOESM1_ESM.docx]

**Icings and groundwater conditions in permafrost catchments of northwestern Canada**

Hugo Crites^1^, Steve V. Kokelj^2^, Denis Lacelle^1,*^

^1^Department of Geography, Environment and Geomatics, University of Ottawa, Ottawa, ON, Canada

^2^Northwest Territories Geological Survey, Government of Northwest Territories, Yellowknife, NWT, Canada

*corresponding author: dlacelle@uottawa.ca

Scientific Reports – Supplementary Online Material

Content:

Tables S1-S2

Figures S1-S5

**Table S1.** 1980-2010 mean annual air temperature (MAAT) and total precipitation at nine meteorological stations in study area. Sen slope value of 1980-2016 trend in mean annual air temperature and total precipitation.

| Site | Latitude | Longitude | MAAT (°) | Total Precipitation (mm) | Sen slope, temperature (° yr^-1^) | Sen slope,  precipitation (mm yr^-1^) |
| --- | --- | --- | --- | --- | --- | --- |
| Faro, YT | 62.21 | -133.37 | -2.2 | 316.1 | 0.03 | -0.65 |
| Fort Liard, NWT | 60.24 | -123.47 | -0.9 | 438.2 | 0.01 | 1.12 |
| Fort Simpson, NWT | 61.76 | -121.24 | -2.7 | 379.6 | **0.06** | -0.08 |
| Inuvik, NWT | 68.35 | -133.48 | -8.3 | 240.6 | **0.12** | 0.76 |
| Mayo, YT | 63.72 | -135.87 | -2.4 | 312.8 | 0.03 | 1.50 |
| Norman Wells, NWT | 65.28 | -126.8 | -5.1 | 294.2 | **0.04** | 0.03 |
| Old Crow, YT | 67.57 | -139.84 | -8.4 | 270.4 | 0.07 | 2.41 |
| Watson Lake, YT | 60.12 | -128.82 | -2.5 | 414.5 | 0.03 | 2.01 |
| Yellowknife, NWT | 62.46 | -114.44 | -4.3 | 286.1 | **0.08** | 0.81 |

Bold values represent Mann-Kendall test significant at < 0.1 level. **Table S2.** Number of images selected for analysis in each WRS1 path/row of Landsat scenes (1985-2017).

| Path_row | Landsat 5 | Landsat 7 |
| --- | --- | --- |
| 51_15 | 16 | 0 |
| 52_15 | 17 | 10 |
| 52_16 | 7 | 8 |
| 53_16 | 16 | 0 |
| 54_14 | 17 | 13 |
| 54_15 | 18 | 12 |
| 54_16 | 0 | 2 |
| 55_14 | 18 | 0 |
| 55_15 | 12 | 0 |
| 55_16 | 10 | 0 |
| 57_13 | 20 | 12 |
| 57_14 | 16 | 11 |
| 57_15 | 9 | 5 |
| 57_16 | 6 | 5 |
| 58_13 | 15 | 0 |
| 58_14 | 10 | 0 |
| 58_15 | 9 | 0 |
| 60_12 | 17 | 12 |
| 60_13 | 17 | 11 |
| 60_14 | 10 | 3 |
| 60_15 | 8 | 5 |
| 62_11 | 0 | 12 |
| 62_12 | 18 | 0 |
| 62_13 | 16 | 0 |
| 62_14 | 11 | 0 |
| 62_15 | 11 | 0 |
| 63_11 | 10 | 15 |
| 63_12 | 20 | 14 |
| 63_13 | 11 | 11 |
| 63_14 | 9 | 8 |
| 63_15 | 6 | 6 |
| 65_12 | 11 | 0 |
| 65_13 | 7 | 0 |
| total | 398 | 175 |


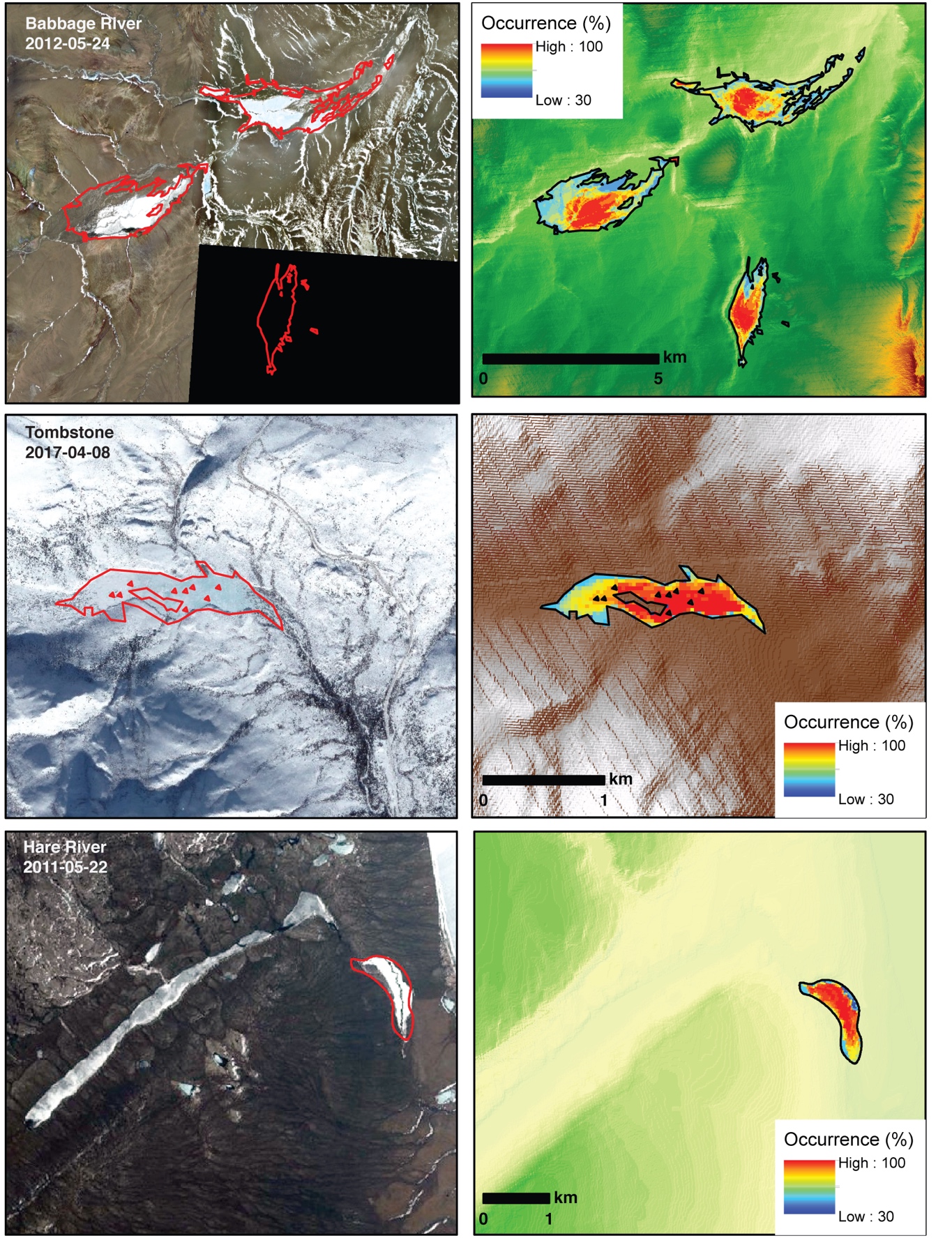


**Figure S1.** Comparison of icings visible from high-resolution spring imageries (panels on the left) with those identified from Landsat image stack (panels on the right). Icing occurrence represents the fraction of years icings were identified between 1985-2017 in the Landsat stack. High-resolution imageries provided by DigitalGlobe Foundation. Map generated using ArcGISv10.

**
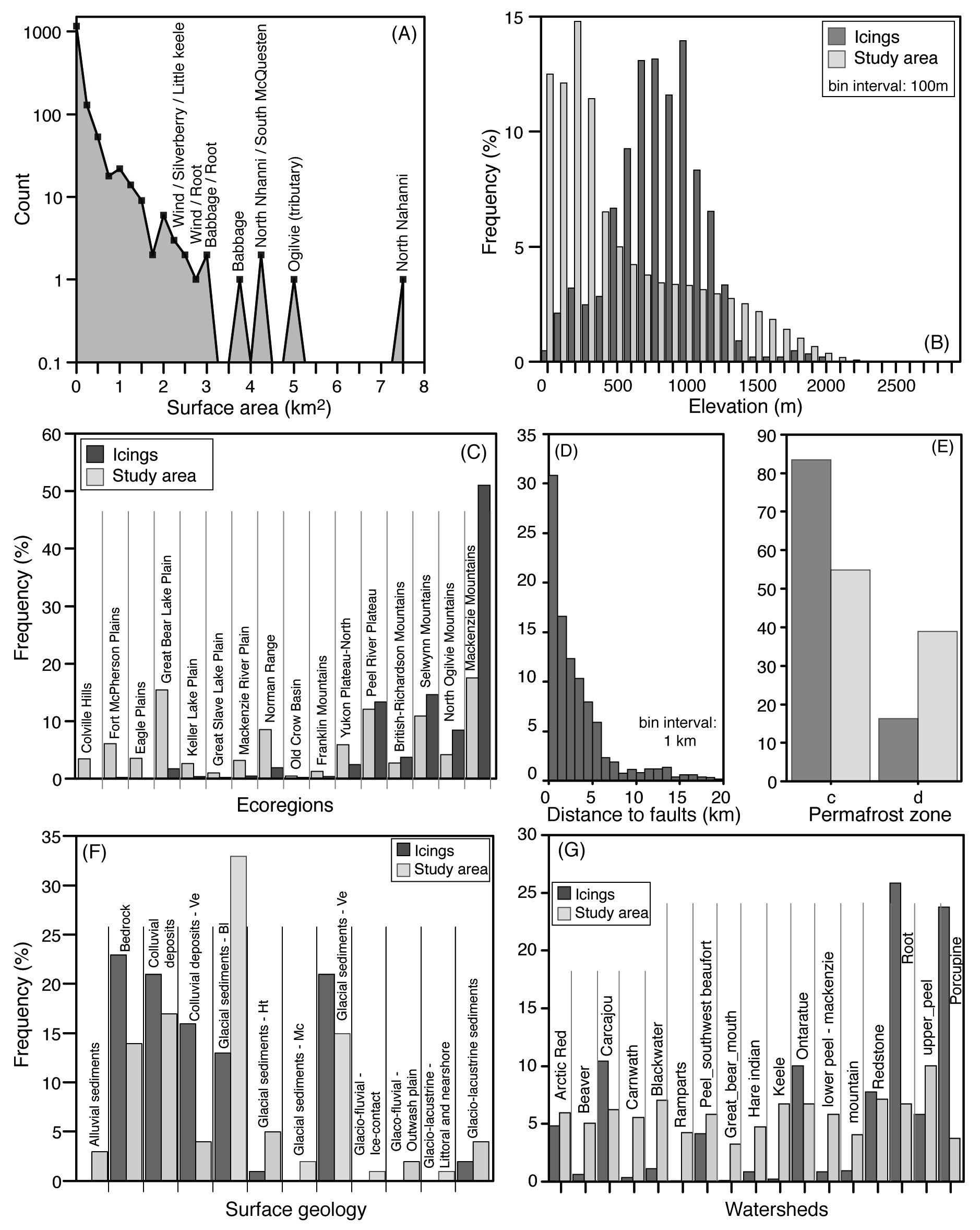
**

**Figure S2 | Frequency distribution of mapped icings and terrain factors in northwestern Canada.** A) Surface area of icings. B) Histogram showing the elevation distribution of icings and terrain in study area in 100 m elevation bins. C) Histogram showing the distribution of icings and ecoregions. D) Histogram showing the distance between icings and the closest geological fault in 1 km bin interval. E) Histogram showing the distribution of icings and permafrost zones. F) Histogram showing the distribution of icings and surficial geology. G) Histogram showing the distribution of icings and watersheds.


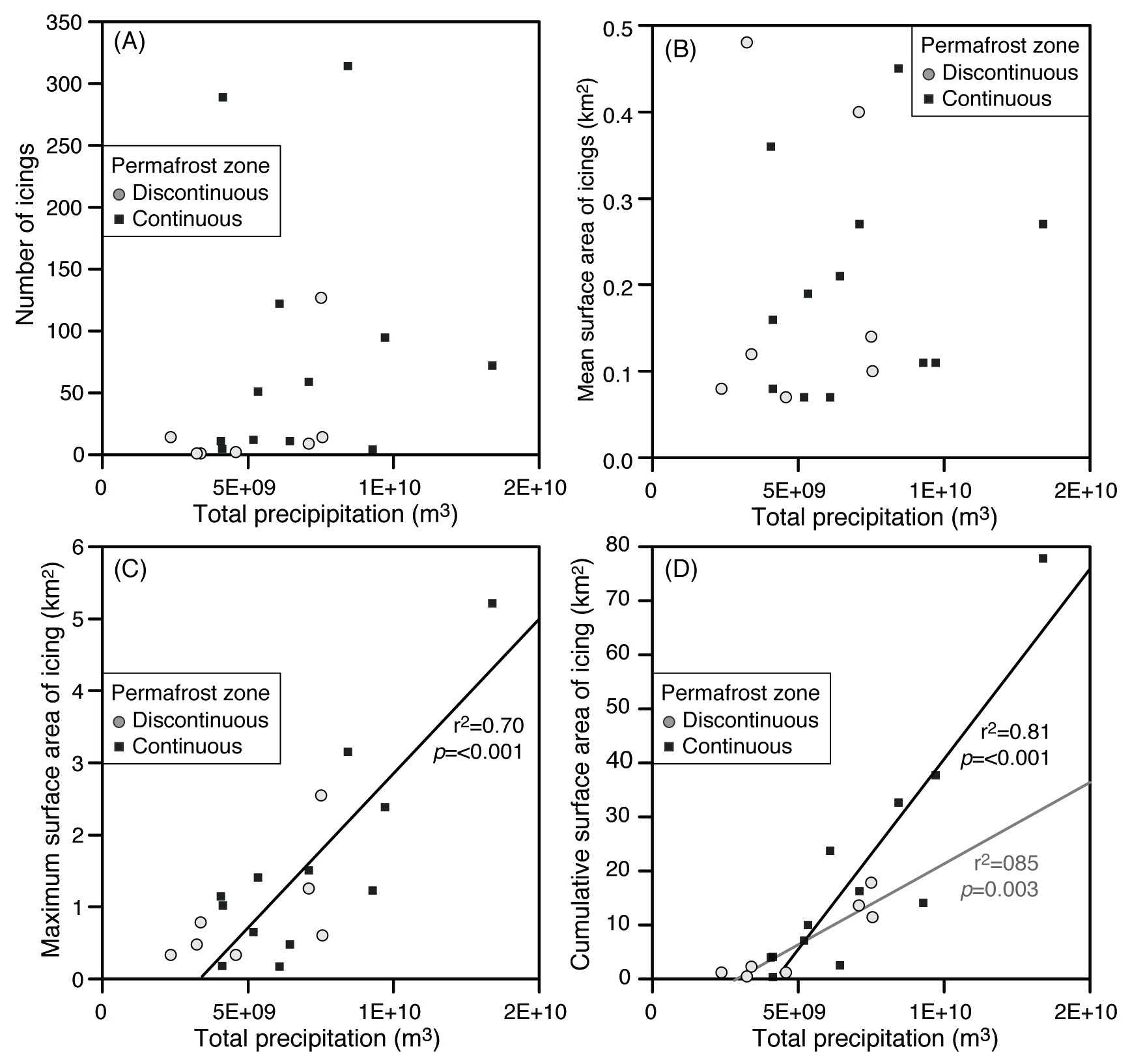


**Figure S3.** Scatter plots of total precipitation in watersheds relative to A) number of icings in the watershed; B) mean surface area of icings in the watershed; C) maximum surface area of icing in the watershed; D) cumulative surface area of icings in the watershed. Linear trend lines are shown for statistically significant relations (*P* < 0.1).


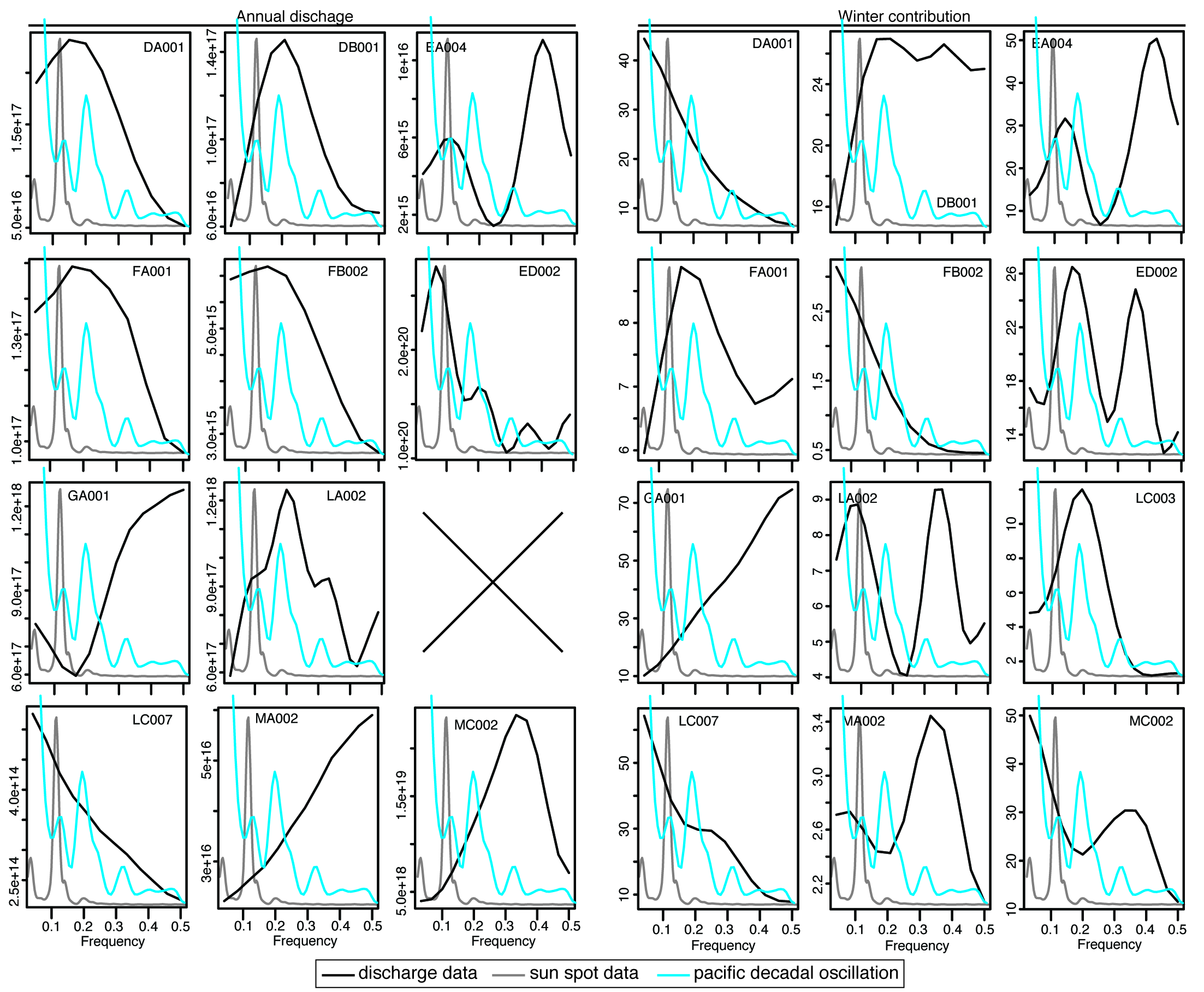


**Figure S4.** Frequency distribution of annual discharge and winter contribution to annual discharge. The x-axis represents frequency in years (1/x).

**
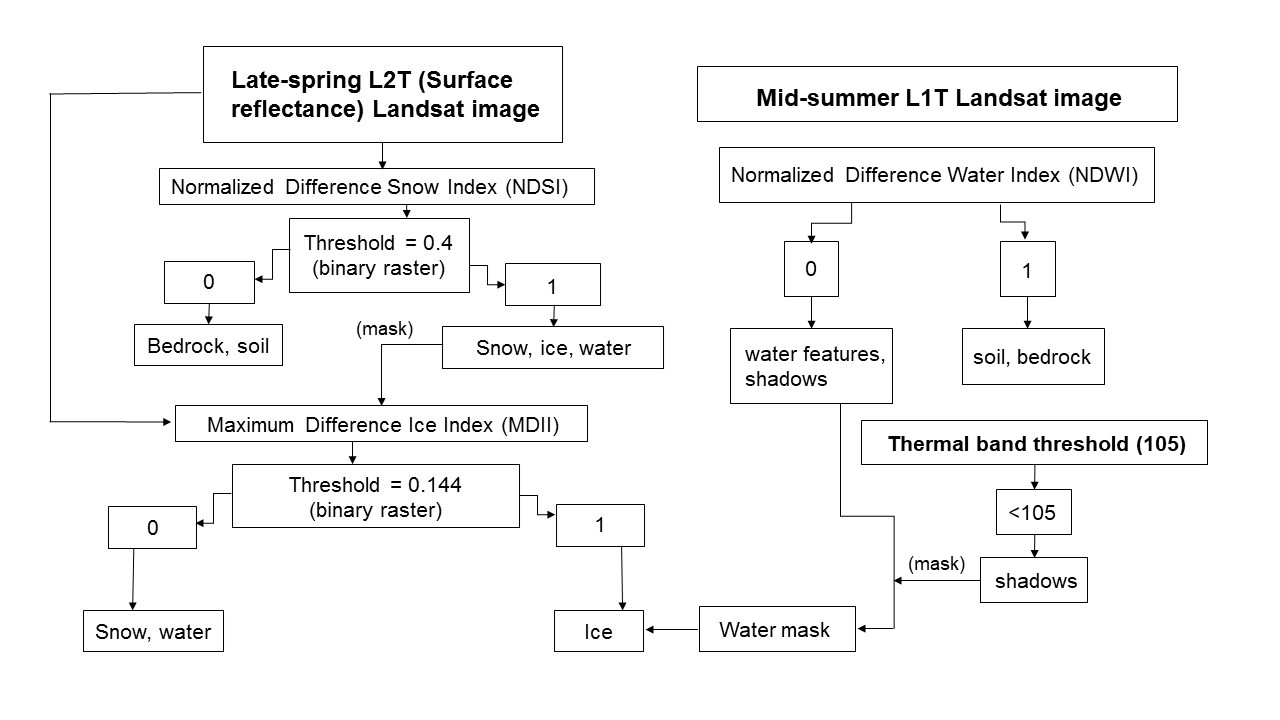
**

**Figure S5.** Flowchart of the image processing methodology modified from ref.^34^ to account for the study area with varied physiographic characteristics.
